# Glyoxalase System in Breast and Ovarian Cancers: Role of MEK/ERK/SMAD1 Pathway

**DOI:** 10.3390/biom14050584

**Published:** 2024-05-15

**Authors:** Muhanad Alhujaily

**Affiliations:** Department of Clinical Laboratory Sciences, College of Applied Medical Sciences, University of Bisha, Bisha 61922, Saudi Arabia; malhujaily@ub.edu.sa; Tel.: +966-176238888

**Keywords:** glyoxalase system, GLO1 and GLO2 enzymes, methylglyoxal (MGO), breast and ovarian cancer

## Abstract

The glyoxalase system, comprising GLO1 and GLO2 enzymes, is integral in detoxifying methylglyoxal (MGO) generated during glycolysis, with dysregulation implicated in various cancer types. The MEK/ERK/SMAD1 signaling pathway, crucial in cellular processes, influences tumorigenesis, metastasis, and angiogenesis. Altered GLO1 expression in cancer showcases its complex role in cellular adaptation and cancer aggressiveness. GLO2 exhibits context-dependent functions, contributing to both proapoptotic and antiapoptotic effects in different cancer scenarios. Research highlights the interconnected nature of these systems, particularly in ovarian cancer and breast cancer. The glyoxalase system’s involvement in drug resistance and its impact on the MEK/ERK/SMAD1 signaling cascade underscore their clinical significance. Furthermore, this review delves into the urgent need for effective biomarkers, exemplified in ovarian cancer, where the RAGE-ligand pathway emerges as a potential diagnostic tool. While therapeutic strategies targeting these pathways hold promise, this review emphasizes the challenges posed by context-dependent effects and intricate crosstalk within the cellular milieu. Insights into the molecular intricacies of these pathways offer a foundation for developing innovative therapeutic approaches, providing hope for enhanced cancer diagnostics and tailored treatment strategies.

## 1. Introduction

Cancer, a formidable adversary to human health, continues to challenge researchers worldwide as they attempt to disentangle its intricate molecular mechanisms [1]. Among the diverse forms of cancer, breast and ovarian cancers stand out for their prevalence and clinical complexity. Breast and ovarian cancers are among the most common types of cancer that affect women worldwide [2]. Despite significant advances in cancer treatment, these cancers remain a significant cause of morbidity and mortality [3]. Recent studies have highlighted the importance of the glyoxalase system and its substrate, methylglyoxal, in cancer progression and therapy [4,5].

The glyoxalase system is a ubiquitous detoxification pathway that converts the highly reactive and toxic compound methylglyoxal to the less toxic compound D-lactate [6]. In cancer cells, the glyoxalase system is upregulated, suggesting its critical role in cancer cell survival and proliferation [7]. The glyoxalase system has been shown to be essential for cancer cell resistance to chemotherapy drugs, such as cisplatin and doxorubicin [8]. The expedition to decipher the underlying molecular details of these malignancies has led researchers to explore novel pathways and mechanisms, including the role of methylglyoxal (MGO) and glyoxal (GO), and the intricate glyoxalase system.

The ubiquitous metabolite methylglyoxal (MGO) has emerged as a molecule of interest due to its dual nature serving as both a metabolic byproduct and a signaling molecule within cellular environments [9]. Studies have implicated MGO in various cellular processes, and its dysregulation has been associated with cancer development [10]. Similarly, glyoxal (GO), another metabolite linked to cellular metabolism, presents a dual role as both a metabolic byproduct and a signaling molecule, adding layers of complexity to its involvement in cancer-related pathways [11].

At the core of the defense against the cytotoxic effects of MGO and GO lies the glyoxalase system, a cellular detoxification pathway crucial for maintaining cellular homeostasis. This intricate system comprises two key enzymes, glyoxalase I and glyoxalase II, each playing distinct roles in the detoxification process [12]. Dysregulation of the glyoxalase system has been implicated in various diseases, including cancer, underscoring its significance in cellular health [13].

Furthermore, the MEK/ERK/SMAD1 signaling pathway, known for its involvement in cellular processes such as proliferation, differentiation, and apoptosis, has gained attention in the context of cancer progression [14]. Understanding the crosstalk between this signaling pathway and the glyoxalase system provides additional layers to the complicated molecular landscape associated with breast and ovarian cancers.

In this review article, we embark on a comprehensive exploration of the molecular intricacies of breast and ovarian cancers, with a focus on the roles of MGO, GO, and the glyoxalase system. By dissecting these elements, we aim to provide a deeper understanding of the underlying mechanisms driving cancer progression. The inclusion of specific discussions on glyoxalase I, glyoxalase II, and the MEK/ERK/SMAD1 signaling pathway adds granularity to our exploration, laying the groundwork for potential therapeutic interventions.

Through this exploration, we aim to not only delineate the molecular intricacies but also to identify potential targets for therapeutic interventions, bringing us one step closer to more effective and targeted treatments for breast and ovarian cancers.

## 2. Methyglyoxal (MGO)

Methylglyoxal (MGO), characterized by its two carbonyl groups, is an active compound that belongs to a group of highly reactive dicarbonyl metabolites, alongside glyoxal (GO) and 3-deoxyglucosone (3-DG) [15]. The human body uses MGO as an essential endogenous dicarbonyl metabolite that is found in many different tissues and organs. Many physiological problems, such as hyperglycemia, renal disorders, and malignant tumors, have been linked to elevated levels of MGO over the normal concentration range [4]. When dicarbonyl metabolites abnormally accumulate and cause increased protein and DNA alteration, this condition is referred to as “dicarbonyl stress”. An imbalance in dicarbonyl metabolites and increased exposure to exogenous dicarbonyls may be the cause of this stress [16].

The main process by which MGO is produced is the non-enzymatic breakdown of dihydroxyacetone phosphate (DHAP) and glyceraldehyde-3-phosphate (G3P) during glycolysis. Hydrolysis, DHAP and G3P dephosphorylation, lipid peroxidation, threonine catabolism, acetone oxidation catalyzed by cytochrome P4502E1, autoxidation of glucose, and glycated protein breakdown are other sources [17]. The in situ concentration of DHAP is usually larger than that of G3P, even though the likelihood of G3P degrading into MGO is higher than that of DHAP. Triosephosphates in both forms are, therefore, necessary for the production of MGO [16,17]. MGO is not solely a byproduct of cell metabolism; it is also obtained through dietary intake, primarily from sources like coffee and other beverages. But before being absorbed, ingested MGO goes through a metabolic process and causes pre-systemic dicarbonyl stress [18].

The glyoxalase system is the main mechanism by which the body gets rid of MGO; aldoketo reductases (AKRs) and aldehyde dehydrogenases (ADHs) metabolize a smaller amount of it [19,20,21]. To stop MGO from glycating, the glyoxalase system functions as an enzymatic defense by changing MGO into hydroxyacetone and pyruvate [22]. In many human tissues, the Glo system’s ability to metabolize MGO is far greater than that of AKRs; however, the renal medulla is an exception, as AKR expression is notably elevated there [23]. MGO is typically generated during glycolysis and processed by the glyoxalase system, which keeps levels low in biological systems. On the other hand, excessive scavenging systems overload and extended ingestion of MGO-rich meals might cause aberrant glycolysis and subsequent buildup of MGO [4]. Advanced glycation end-products (AGEs), a category of MGO-derived adducts, modify lipids, the free amino groups of proteins, and nucleic acids. These modifications cause major cytotoxicity and tissue damage that are linked to MGO-related metabolic diseases [24].

**Figure 1:**
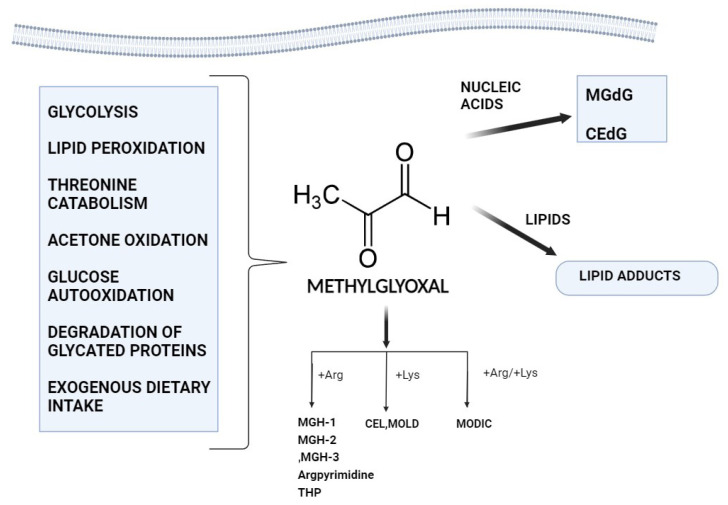
A visual primer on how methylglyoxal forms and works in the body.

Most of the MGO nucleotide adducts, including MGdG, are formed mainly by the interaction of MGO with deoxyguanosine. These adducts have the potential to cause malignant transformations and are mutagenic. Moreover, argpyrimidine, tetrahydropyrimidine, and MGO-derived hydroimidazolones (MGO-H1, MGO-H2, and MGO-H3) are formed as a result of MGO’s irreversible interaction with arginine [25]. Though less so than arginine, MGO can also alter lysine residues to generate Nϵ-(1-carboxyethyl) lysine (CEL) and 1,3-di(Nϵ-lysino)-4-methyl-imidazolium (MOLD). An adduct known as MODIC is created when MGO reacts with arginine and lysine alike. Furthermore, MGO results in long-lasting lipid alterations [26] (Figure 1).

Moreover, the generated AGEs have the ability to attach to the receptor for AGEs (RAGE), which mediates signal transduction and promotes the production of intracellular reactive oxygen species (ROS) [27]. RAGE signaling activity is linked to a number of cellular alterations, such as oxidative stress and inflammation. It also plays a part in carcinogenesis and increases apoptosis and anoikis, two processes that lead to cell death. Tumor growth and metastasis are known to be inhibited by reduced RAGE expression [28].

## 3. Glyoxal (GO)

Glyoxal (GO), a dicarbonyl metabolite, is intricately involved in cellular metabolism and signaling. As part of a group of highly reactive dicarbonyl compounds, including methylglyoxal (MGO) [15], GO plays a crucial role in various physiological and pathological processes. Elevated levels of GO are associated with conditions such as hyperglycemia, kidney diseases, and cancer, contributing to dicarbonyl stress [29].

GO is produced endogenously during glycolysis through the degradation of glyceraldehyde-3-phosphate (G3P) and dihydroxyacetone phosphate (DHAP) [30]. Additionally, it can be obtained from dietary sources [31]. The glyoxalase system, consisting of glyoxalase I and glyoxalase II, emerges as a central defense mechanism against GO-induced cytotoxicity. This enzymatic system efficiently metabolizes GO, preventing its accumulation within cells [32]. The dysregulation of GO metabolism leads to dicarbonyl stress, resulting in modifications to nucleic acids, proteins, and lipids. GO exhibits reactivity with proteins and DNA, forming advanced glycation end-products (AGEs) [33]. Its interaction with arginine residues in proteins and deoxyguanosine (dG) in DNA leads to increased mutations and decreased DNA replication. Notably, GO-induced modifications are linked to specific mutation sites, adding a layer of complexity to its impact on genetic stability [34].

The detoxification of GO primarily occurs through the glyoxalase system, with glutathione (GSH)-dependent glyoxalases (Glo1 and Glo2) catalyzing the conversion of GO to S-2-hydroxyethylglutathione [35]. Additionally, glyoxalase 3 (Glo3) can metabolize GO independently, albeit without cofactors [11]. The consequences of GO exposure extend to the activation of receptors for AGE (RAGE) signaling pathways. This activation triggers cellular changes, inflammation, and oxidative stress. The intricate interplay between GO and pathways associated with cancer is evident through the association of RAGE expression with tumor development, highlighting the potential involvement of GO in carcinogenesis [36,37]. In summary, GO’s versatility in cellular metabolism, its intricate interactions with biomolecules, and its role in dicarbonyl stress underscore its significance in health and disease. Understanding the nuances of GO metabolism and its implications provides a foundation for targeted therapeutic interventions, particularly in diseases like cancer, where its influence on cellular processes is pronounced.

## 4. Glyoxalase System

The glyoxalase system, an essential enzymatic pathway present in all human cells, has been a subject of interest in the context of cancer research since its introduction by Dakin and Dudley in 1913 [8]. This complicated system plays a crucial role in detoxifying methylglyoxal (MGO), a compound produced as a byproduct of glycolysis [34]. Elevated levels of MGO, often associated with increased glycolytic activity in cancer cells, contribute to the formation of advanced glycation end-products (AGEs) and oxidative stress, fostering a microenvironment conducive to tumorigenesis [38]. The glyoxalase system comprises two enzymes: S-lactoylglutathione lyase, referred to as GLO1 (EC 4.4.1.5), and hydroxyacylglutathione hydrolase, known as GLO2 (EC 3.2.1.6). Additionally, a catalytic amount of GSH is also present in this system.

**Figure 2:**
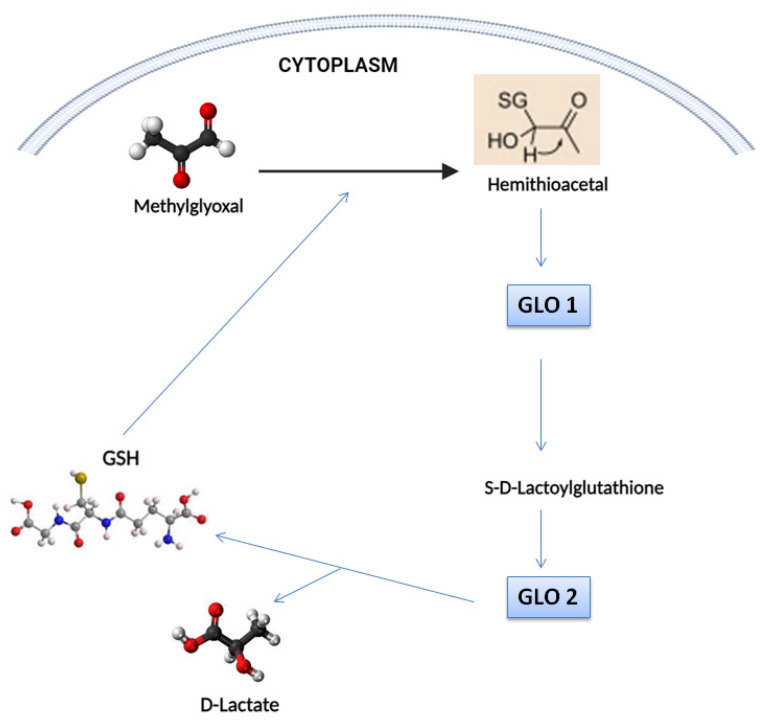
Glyoxalase system: enzymatic detoxification of methylglyoxal (MGO).

Central to the glyoxalase system are the enzymes glyoxalase I (GLO1) and glyoxalase II (GLO2), working in tandem to neutralize the cytotoxic effects of MGO. GLO1 catalyzes the conversion of MGO to S-D-lactoylglutathione, utilizing reduced glutathione (GSH) as a cofactor. This reaction is pivotal in preventing MGO-induced damage to cellular components [39]. Subsequently, GLO2 hydrolyzes S-D-lactoylglutathione to produce D-lactate and regenerate GSH, completing the detoxification cycle [40] (Figure 2).

In the context of cancer, the dysregulation of the glyoxalase system has been observed. Altered expression levels of GLO1 have been reported in various cancer types, with increased expression potentially serving as an adaptive response to counteract MGO-induced damage. Conversely, decreased GLO1 expression has been associated with more aggressive cancer phenotypes, suggesting a complex role for the glyoxalase system in cancer progression [41].

Researchers are exploring the therapeutic potential of targeting the glyoxalase system in cancer treatment. Modulating GLO1 activity has emerged as a strategy to sensitize cancer cells to existing therapies or induce direct cytotoxic effects. The glyoxalase system’s association with cancer is underscored by its role in detoxifying MGO, a byproduct of glycolysis often elevated in cancer cells due to increased glycolytic activity [7]. The dysregulation of Glo1 expression has been observed in various cancer types, with both increased and decreased expression associated with specific cancer phenotypes [42]. Researchers are actively exploring therapeutic strategies targeting the glyoxalase system in cancer treatment. Modulating Glo1 activity is considered a potential approach to sensitize cancer cells to existing therapies or induce direct cytotoxic effects [43]. The system’s involvement in cellular defense against oxidative stress is particularly relevant in cancer, where oxidative stress is a hallmark feature, and preventing MGO-induced oxidative damage contributes to cellular resilience in the tumor microenvironment [44].

Understanding the complex involvement of the glyoxalase system in cancer biology provides valuable insights into cellular survival mechanisms and potential vulnerabilities. As research progresses, further exploration of the glyoxalase system’s role in cancer could lead to innovative therapeutic strategies aimed at disrupting cancer cell survival mechanisms and improving overall treatment outcomes.

## 5. Glyoxalase 1

The human GLO1 gene, located on chromosome 6p21.2 between HLA and the centromere, plays a crucial role in cellular function, and its genetic makeup reveals a story rich in diallelic diversity [45]. In individuals with heterozygosity, the gene produces a dimeric protein weighing 42 kDa (by sequence) or 46 kDa (per gel filtration) with an isoelectric point (pI) dancing between 4.8 and 5.1. This genetic tale unfolds through three GLO1 variants—GLO1-1, GLO1-2, and GLO2-2—illustrating the symphony of homozygous or heterozygous expression on an autosomal locus [39,46,47]. GLO1 operates as a metal-dependent maestro, carrying a Zn^2+^ ion per subunit in *E. coli* or a Ni^2+^ ion in humans. The orchestration involves regulatory players such as AP-2α, E2F4, NF-κB, and AP-1, coupled with elements like ARE, MRE, and IRE. The narrative extends to GLO1’s exon 1, where a dance occurs with Nrf2. While Nrf2 activators like sulforaphane and resveratrol are implicated as GLO1 conductors, the precise details of this regulatory composition remain unexplored [48].

Glo1 expression is controlled by a complex network of regulatory components, post-translational enzyme modifications, and variations in gene expression [49]. Phosphorylation, nitrosylation, and glutathionylation are examples of post-translational changes. AP-2a, E2F4, NF-kB, and Nrf2 all aid in positive regulation by strengthening the Glo1 promoter. Glo1 is induced by Nrf2 activators such as resveratrol and sulforaphane [50]. The coupling of MGOs to Kelch-like ECH-associated protein 1 (Keap1) can impair the nuclear translocation of Nrf2, which is essential for Glo1 activation, suggesting a possible detoxifying pathway [51] (Table 1).

In contrast, HIF1a, RAGE, and the struggle between Nrf2 and the NF-kB system engaged in inflammation adversely influence Glo1 expression [52]. The human genome’s copy number variation (CNV) of the Glo1 gene enables higher expression in the healthy population through low-level duplication. Many human cancers have been shown to have an increased Glo1 copy number; breast cancer, sarcomas, and non-small cell lung cancer have the greatest frequencies of these tumors [53,54]. It has been shown that there is a connection between an increase in Glo1 copy number and a bad prognosis for gastric malignancies. This finding provides insight into the possible consequences for therapy choices and disease prognosis [43,55].

## 6. Glyoxalase 2

GLO2, situated at 16p13.3, takes center stage in the glyoxalase narrative, encoded by the hydroxyacylglutathione hydrolase gene (HAGH). The script of human GLO2 unfolds across 10 exons, producing two distinct mRNAs that craft the cytosolic GLO2 (28.8 kDa) and the mitochondrial GLO2 (33.9 kDa) [56]. Both forms share an isoelectric point of 8.3, showcasing their molecular dance with efficiency in hydrolyzing S-D-lactoylglutathione [57].

This enzymatic maestro, adorned with a metallo-β-lactamase-like and α-helical domain, hosts a Fe (II) Zn (II) center [58]. The active site, set in two domains, witnesses the graceful pirouette of Fe (II), influencing neither the catalytic prowess nor the efficiency of GLO2. In the genetic libretto, intron 1 of human GLO2 bows with a p53-response element, activated by p63 and p73 to orchestrate an increase in GLO2 expression [59]. Recent evidence highlights Glo2’s independent role in some malignant cells, possibly nonenzymatic. In certain contexts, Glo2 contributes to proapoptotic effects, such as in non-small cell lung cancer A549 cells, after Oleuropein treatment to induce apoptosis [60]. However, in prostate cancer cells, Glo2 takes on an antiapoptotic nonenzymatic role, stimulating cell proliferation and eluding apoptosis through mechanisms dependent on the androgen receptor and involving the p53-p21 axis [61]. This emerging dual role adds a layer of complexity to our understanding of Glo2 in cancer biology (Table 2).

## 7. MEK/ERK/SMAD1 Signaling

The MEK/ERK/SMAD1 signaling pathway emerges as a critical player in the complicated landscape of cancer biology, exerting profound influences on tumorigenesis, tumor growth, invasion, and metastasis [62]. This signaling cascade involves the activation of Mitogen-Activated Protein Kinase (MEK) and Extracellular Signal-Regulated Kinase (ERK), along with the SMAD1 protein within the transforming growth factor-beta (TGF-β) pathway [63]. Understanding the multi-layered roles of MEK/ERK/SMAD1 signaling in cancers is pivotal for unraveling the complexities of this disease and developing targeted therapeutic strategies (Table 3).

One of the hallmark features of cancer is uncontrolled cell growth and proliferation. The MEK/ERK pathway, when aberrantly activated, is frequently implicated in driving these processes [63]. Through a series of phosphorylation events, MEK activates ERK, which subsequently regulates key cellular processes involved in cell cycle progression. The interplay between SMAD1 and the MEK/ERK axis in orchestrating these events is an area of active research [64]. Studies suggest that SMAD1, a downstream effector in the TGF-β pathway, may interact with MEK/ERK signaling to fine-tune cell proliferation in specific cancer contexts, adding an additional layer of complexity to the regulation of tumor growth [65].

Epithelial–Mesenchymal Transition (EMT) is a pivotal phenomenon in cancer progression, endowing cancer cells with enhanced migratory and invasive capabilities. The MEK/ERK pathway has been implicated in inducing EMT, facilitating the transition of cancer cells from a stationary epithelial state to a motile mesenchymal state [66]. Concurrently, SMAD1, as part of the TGF-β pathway, plays a role in EMT regulation. The crosstalk between MEK/ERK and SMAD1 in governing EMT underscores the intricate regulatory networks that contribute to the metastatic potential of cancer cells [67].

Metastasis, the spread of cancer cells from the primary tumor to distant sites, is a complex and orchestrated process. MEK/ERK signaling has been associated with promoting tumor invasion by influencing cell adhesion, extracellular matrix remodeling, and cytoskeletal changes [68]. Through its downstream effectors, ERK can modulate the expression of genes involved in these processes, contributing to the invasive behavior of cancer cells. SMAD1, interacting with other SMAD proteins in the TGF-β pathway, may enhance metastasis by regulating genes associated with cell motility [69]. The coordinated actions of MEK/ERK and SMAD1 shed light on the interconnected pathways that facilitate the dissemination of cancer cells throughout the body [70].

Angiogenesis, the formation of new blood vessels, is a hallmark of cancer progression, as it provides tumors with a nutrient supply [71]. The MEK/ERK pathway has been implicated in angiogenesis, with ERK influencing the expression of angiogenic factors. SMAD1, in the context of TGF-β signaling, contributes to angiogenesis regulation, further highlighting the convergence of these pathways in shaping the tumor microenvironment [72]. The orchestrated interplay between the MEK/ERK/SMAD1 signaling pathways influences the intricate balance between proangiogenic and antiangiogenic factors, ultimately impacting the vascularization of tumors [73] (Table 4).

Therapeutically targeting components of the MEK/ERK pathway has emerged as a promising strategy in cancer treatment. Small-molecule inhibitors designed to block MEK or ERK activity have shown efficacy in preclinical and clinical studies, demonstrating the therapeutic potential of disrupting this pathway [74]. Additionally, the TGF-β pathway, including SMAD proteins, is under scrutiny as a potential target for cancer therapy [75]. The crosstalk and convergence of the MEK/ERK and SMAD1 signaling pathways present an intriguing avenue for developing combination therapies that effectively disrupt multiple nodes in the intricate signaling network fueling cancer progression [76].

However, the context-dependent effects of MEK/ERK/SMAD1 signaling in cancers add a layer of complexity to therapeutic interventions [14]. The response to targeted therapies can vary widely between different cancer types and even within subtypes of the same cancer. The tumor microenvironment, genetic mutations, and the intricate crosstalk with other signaling pathways contribute to this context-dependent behavior [77]. Research efforts are focused on deciphering the molecular intricacies that dictate the divergent outcomes of MEK/ERK/SMAD1 signaling in various cancer contexts [78].

Recent studies have shed light on the profound impact of MEK/ERK/SMAD1 signaling in the context of specific cancers, offering insights into the molecular mechanisms that drive tumorigenesis [79,80]. Prostate cancer, for example, provides an intriguing backdrop for exploring the involvement of this signaling cascade [81]. PTEN loss, a common occurrence in prostate cancer, heralds the upregulation of both GLO1 and GLO2 within the glyoxalase system. This rise in glyoxalase enzymes is facilitated by the activation of the PI3K/AKT/mTOR pathway through the PI3K/AKT/mTOR/p-PKM2(Y105)/ERa axis, ultimately propelling prostate cancer progression [82]. The intricate interplay between MEK/ERK/SMAD1 signaling and the glyoxalase system in prostate cancer underscores the complexity of the molecular networks governing cancer phenotypes [83].

Furthermore, the role of Glo2, a component of the glyoxalase system, extends beyond its enzymatic functions. Emerging evidence suggests a novel independent role of Glo2 in certain malignant cells, possibly in a nonenzymatic manner. In non-small cell lung cancer cells, Glo2 is implicated in the proapoptotic effects of Oleuropein [56]. Interestingly, Oleuropein increases mitochondrial Glo2 protein expression levels without enhancing the enzyme’s activity, highlighting a nonenzymatic function. This unconventional role of Glo2 is context-dependent, as it exhibits an antiapoptotic, nonenzymatic function in prostate cancer cells [84]. This duality in Glo2’s function underscores the intricate interplay between enzymatic and nonenzymatic roles within the glyoxalase system, influenced by the specific cellular context [13].

A recent study investigating the impact of glyoxalase 1 (GLO1) silencing on breast cancer cells adds another layer to our understanding of cancer biology. Elevated aerobic glycolysis, characteristic of malignant transformation, results in increased methylglyoxal (MGO) production [4]. GLO1, a crucial enzyme in the glyoxalase system, detoxifies MGO. Silencing GLO1 leads to endogenous dicarbonyl stress and enhanced growth and metastasis in breast cancer models [7]. The study uncovered a pro-metastatic signature related to dicarbonyl stress in breast cancer cells. The MEK/ERK/SMAD1 pathway was hyperactivated under MGO stress conditions, connecting this pathway to a pro-metastatic phenotype [85]. High-throughput transcriptome profiling highlighted novel connections between MGO dicarbonyl stress, ECM remodeling, and enhanced cell migration [79]. The study showcased the functional link between MEK/ERK/SMAD1 cascade activation and the acquisition of a pro-metastatic phenotype in breast cancer cells. The convergence of MEK/ERK/SMAD1 signaling with other crucial systems, such as the glyoxalase system, further highlights the interconnected nature of cellular processes in cancer [14]. Continued research in this field holds the promise of uncovering novel therapeutic targets and refining treatment approaches for a diverse array of cancers, ultimately advancing precision medicine in oncology (Table 5).

## 8. Breast Cancer

**Figure 3:**
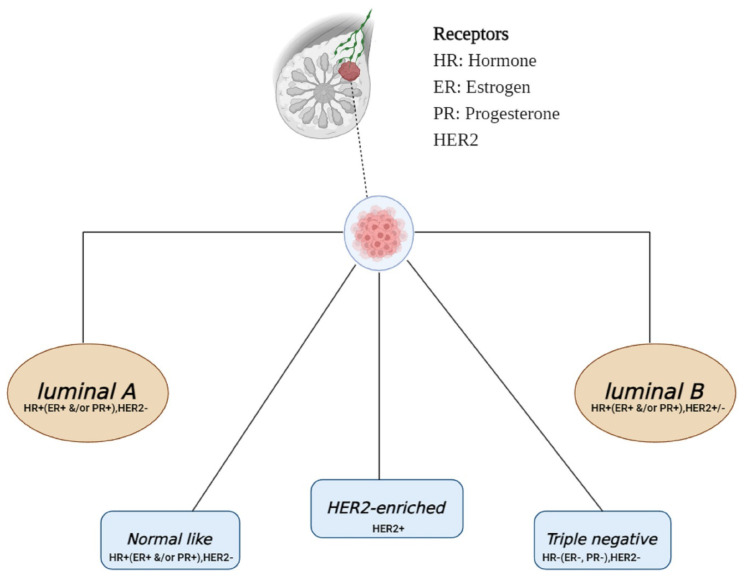
A breast cancer schematic categorizes subtypes based on hormone receptor expression, Ki-67, and HER2 status. Luminal A, the common subtype, has low Ki-67. HER2-enriched tumors show HER2 overexpression, growing faster than luminal types. Triple-negative tumors, most aggressive and frequent in younger patients, lack ER, PR, and HER2. Abbreviations: ER (estrogen receptor), PR (progesterone receptor), HER2 (epidermal growth factor receptor 2).

Breast cancer is the most common cancer in women globally, accounting for 2.09 million new cases and 0.6 million deaths every year [86]. Breast cancer’s intrinsic subtypes, identified by the expression of the proliferation marker Ki-67, human epidermal growth factor receptor 2 (HER2), progesterone receptor (PR), and estrogen receptor (ER), reveal the intricacy of the disease [87] (Figure 3).

Therapeutic strategies are intricately woven around these molecular markers, leading to tailored treatments for ER-positive or PR-positive cases with anti-endocrine therapy and HER2-positive cases benefiting from monoclonal antibodies [88]. In contrast, triple-negative breast cancer, lacking these receptors, poses a significant challenge with no targeted therapy available, necessitating reliance on chemotherapy [89]. However, the clinical classification based on immunohistochemistry serves as a surrogate, lacking the depth of gene expression analysis and failing to unveil all internal molecular intricacies [90].

The Investigation of Glo1’s function in breast cancer began in 2001 with A. Rulli et al.’s study of Glo1’s particular activity in breast carcinoma and healthy mammary gland tissue. The study, conducted on samples drawn from 20 women between 1999 and 2000, revealed a significant upregulation of Glo1 in tissues and cells of human breast cancer [91]. This upregulation, validated through spectrophotometrical assays and electrophoretic patterns, underscored the potential Glo1’s function in breast cancer [92].

Beyond the biochemical evidence, Glo1’s involvement in breast cancer was substantiated by its promotion of cell proliferation, invasion, and migration, coupled with the suppression of apoptosis [7]. As Glo1 overexpression was linked to aggressive clinicopathological traits such as lymph node metastases, lymphovascular invasion, a greater tumor grade, and an advanced TNM stage, the clinical implications of Glo1 became apparent [93]. Glo1 overexpression was found to be independently related to lower overall survival and recurrence-free survival, which highlights its critical involvement in the course of breast cancer [94] (Table 6).

According to complementary investigations, oxidative stress (NFE2L2) and inflammation (NF-kB) in malignant breast cells are closely related to the regulation of Glo1 mRNA [94]. The study by Guo et al. illuminated a possible therapeutic option by demonstrating how inhibiting Glo1 could prevent the growth of breast cancer by downregulating MMP-9 and Bcl-2 and altering the MAPK signaling pathway [95]. Nonetheless, a thorough understanding of the fundamental molecular biology and mechanisms behind breast cancer remains necessary.

Beyond its association with tumorigenesis, Glo1 emerges as a key player in drug resistance, a major hurdle in effective tumor treatment. Chemotherapeutic resistance, particularly to doxorubicin, has been linked to Glo1 upregulation. In the quest to overcome this challenge, Glo1 inhibitors have shown promise in reversing drug resistance. Troglitazone, a thiazolidinedione, demonstrated downregulation of Glo1 expression, reinstating sensitivity to doxorubicin [96]. The predictive potential of Glo1 abundance in radiotherapy outcomes further emphasizes its role in treatment response. Overexpression of Glo1 was correlated with a shorter relapse-free survival after radiotherapy [94]. As more than 50% of tumor treatment drugs originate from natural compounds, the exploration of bioactive compounds like resveratrol, curcumin, and piperine as Glo1 inhibitors opens avenues for novel therapeutic interventions [97,98,99,100].

Distant metastasis, responsible for approximately 90% of cancer-associated mortality in breast cancer, introduces the imperative need to identify key regulators in this intricate process [101]. Recent reports propose a cooperative involvement of Glo1 and PKCλ in breast cancer progression. High expression levels of both Glo1 and PKCλ were associated with a worse prognosis in patients with stage III–IV breast cancer. The inhibitors of Glo1 (TLSC702) and PKCλ (aurothiomalate) emerge as potential pharmacological approaches to manage late-stage breast cancer by suppressing cell viability and tumor-sphere formation [102].

A noteworthy finding in Marie-Julie Nokin et al.’s study revealed Glo1’s tumor-suppressive function in breast cancer cells [79,103]. Not only did silencing Glo1 lead to increased levels of methylglyoxal (MGO), but it also greatly raised the burden of lung tumors and encouraged tumor development and metastasis in vivo. The complex nature of Glo1’s activity in breast cancer is highlighted by its dualistic role, which can act as a suppressor or a promoter depending on the situation [104] (Table 7).

Seemingly contradictory data regarding MGO’s effect on cancer cells, defined by low-dose stimulation and high-dose inhibition of tumor metastasis, necessitate a nuanced understanding [105]. Determining MGO concentrations becomes imperative when applying Glo1 inhibitors, highlighting the importance of context-dependent responses in unraveling the complexities of breast cancer biology.

In conclusion, the saga of Glo1 in breast cancer unfolds as a multifaceted narrative, weaving through tumorigenesis, drug resistance, prognostic challenges, and metastasis. The intricate interplay of Glo1 with molecular pathways and its dualistic role underscores the need for further research to unveil its complete biological portrait, offering novel therapeutic targets and refining treatment approaches in the relentless battle against breast cancer.

## 9. Epithelial Ovarian Cancer

Ovarian cancer, while less common than other cancers, is the most fatal of the female reproductive tract malignancies in the United States [106]. The lack of adequate screening procedures frequently leads to late-stage diagnosis, in which malignancies have diffused and have a bad prognosis [107]. The conventional treatment for ovarian cancer is cytoreductive surgery followed by postoperative adjuvant chemotherapy [108]. Unfortunately, even in countries with advanced medical technology, such as the United States and Canada, the five-year survival rate remains approximately 47%, owing to delayed diagnosis, recurrence, and chemoresistance [109].

Pathological biopsy is now the gold standard for diagnosing ovarian cancer, and early screening techniques have drawbacks [110]. Although human epididymis protein 4 and carbohydrate antigen 125 are examples of biomarkers that have shown promise in screening, their limited sensitivity and specificity prevent them from being widely used [111]. This emphasizes how critical it is to find new biomarkers for ovarian cancer early detection (Table 8).

In view of increasing therapeutic targets, researchers have focused on the Receptor for Advanced Glycation End Products (RAGE)-ligand pathway, which has been identified as a novel target for certain cancer therapies [112,113]. RAGE was found to be upregulated in ovarian cancer tissue when compared to comparable normal tissue. A substantial link has been shown between high RAGE expression levels and unfavorable clinicopathological parameters such as the tumor size, the depth of stromal invasion, lymphovascular invasion, and the tumor stage, indicating that RAGE plays an important role in the course of ovarian cancer [114]. The study’s area under the curve value for RAGE was 0.86, showing that RAGE mRNA levels have a relatively high sensitivity and specificity for distinguishing between malignant and non-malignant tissues. This shows that RAGE overexpression could be used as a biomarker to diagnose ovarian cancer, a conclusion reinforced by consistent findings by Poljicanin et al. [115].

Examining the molecular causes of ovarian cancer in greater detail reveals that the majority of ovarian cancers start from a single layer of surface epithelial cells (OSE), which make up a very small percentage of the entire ovarian mass [116]. Normal OSE cells from women with a family history of breast and ovarian cancer show distinct abnormalities from those without that history. Proteomes from SV-40-transformed FH-OSE cell lines and control OSE lines were analyzed by Smith Beckerman, who found that FH-OSE cells expressed higher levels of glyoxalase 1 (Glo1) and other proteins [117]. This implies that the development and spread of ovarian cancer are linked to elevated Glo1 expression (Table 9).

Despite the fact that ovarian cancers are highly curable in their early stages, more than 70% of cases are not identified until the tumor has grown to an advanced stage. This stresses the increased risk of morbidity and mortality associated with advanced-stage illness [118]. Monica Brown Jones’ research discovered a significant level of Glo1 overexpression in invasive ovarian cancers compared to low-malignant potential ovarian tumors. Her research, which used laser capture microdissection and two-dimensional gel electrophoresis techniques, reveals that Glo1 could be a viable early detection marker and treatment target specific to the invasive phenotype [119].

The exact mechanisms of Glo1 in ovarian cancer remain unknown, presenting an intriguing avenue for future research. The potential role of Glo1 as a therapeutic target adds another layer of complexity to its investigation. Encouragingly, more investigations are warranted to provide reliable data and unlock the mysteries surrounding Glo1 in ovarian cancer. In brief, ovarian cancer poses diagnostic challenges and demands a multifaceted approach to unravel its intricacies. From the exploration of novel biomarkers like RAGE to the potential therapeutic implications of Glo1, ongoing research offers hope for improved diagnostic methods and targeted treatments, aiming for better outcomes in the battle against ovarian cancer.

## 10. Conclusions

This investigation into the intrinsic molecular mechanisms involving the glyoxalase system and methylglyoxal in breast and ovarian cancer reveals a complex landscape with profound implications for therapeutic strategies. The dysregulation of the glyoxalase system and the subsequent accumulation of methylglyoxal emerge as significant contributors to cancer progression, influencing key cellular processes.

The therapeutic avenues presented, such as the augmentation of the glyoxalase system and the development of methylglyoxal scavengers, open promising avenues for targeted interventions. The potential synergy with existing cancer therapies suggests a multifaceted approach that could enhance treatment outcomes. Nevertheless, the journey from bench to bedside faces challenges, including the identification of reliable biomarkers, effective clinical translation, and the aspiration for personalized therapeutic regimens.

This exploration of intrinsic molecular mechanisms not only enriches our comprehension of cancer biology but also lays the groundwork for innovative and precise therapeutic interventions. The aspiration is that these insights will transcend the laboratory realm, shaping a new era in cancer treatment characterized by enhanced efficacy and patient-specific approaches. Collaborative efforts across scientific, clinical, and pharmaceutical domains remain paramount in realizing the transformative potential of these findings, offering renewed optimism for individuals grappling with the complexities of breast and ovarian cancer.

## Figures and Tables

**Table 1:** Regulation of Glo1 expression in human cells: an organized summary of the regulatory elements involved in Glo1 expression, along with their roles and associated factors.

Regulatory Element	Role in Regulation
AP-2a	Positive transcriptional regulator [50].
E2F4	Positive transcriptional regulator [50].
NF-kB	Positive transcriptional regulator [50].
AP-1	Positive transcriptional regulator [49].
ARE (Antioxidant Response Element)	Positive transcriptional regulator [49].
MRE (Metal Response Element)	Positive transcriptional regulator [49].
IRE (Iron Response Element)	Positive transcriptional regulator [49].
Phosphorylation	Post-translational modification [50].
Nitrosylation	Post-translational modification [50].
Glutathionylation	Post-translational modification [50].
Nrf2 activators (e.g., sulforaphane, resveratrol)	Positive regulators of Glo1 [50].
MGOs to Keap1	Disrupts Nrf2 nuclear translocation [51].
HIF1a	Negative transcriptional regulator [52].
RAGE	Negative transcriptional regulator [52].
NF-kB activation in inflammation	Negative transcriptional regulator [52].
Copy Number Variation (CNV)	Increased expression with few copies made in healthy populations [53,54].
Increased Glo1 copy number	Observed in various human tumors, highest prevalence in breast cancer, sarcomas, and non-small cell lung cancer [43,55].
Correlation with poor survival	Noted in gastric cancers, suggesting potential implications in disease prognosis and therapeutic interventions [43,55].

**Table 2:** Key features of GLO2 in cancer biology.

Feature	Description
Chromosomal Location	16p13.3 [56].
Gene	Hydroxyacylglutathione hydrolase gene (HAGH) [56].
Exons	10 [56].
mRNA Variants	Cytosolic GLO2 (28.8 kDa) and Mitochondrial GLO2 (33.9 kDa) [56].
Isoelectric Point	8.3 [56].
Catalytic Activity	Efficient hydrolysis of S-D-lactoylglutathione [57].
Protein Domains	Metallo-β-lactamase-like and α-helical domains [57].
Metal Center	Fe (II) Zn (II) center [58].
Active Site	Set in two domains; Fe (II) undergoes graceful movements without affecting catalytic prowess [59].
Genetic Regulation	Intron 1 hosts a p53-response element activated by p63 and p73, orchestrating an increase in GLO2 expression [59].
Dual Role in Cancer	Context-dependent enzymatic and nonenzymatic roles in cancer cells contribute to both proapoptotic and antiapoptotic effects [61].
Proapoptotic Effects (Example)	After Oleuropein treatment, GLO2 induces apoptosis in non-small cell lung cancer A549 cells [60].
Antiapoptotic Effects (Example)	In prostate cancer cells, the nonenzymatic role stimulates cell proliferation and evades apoptosis through the androgen receptor and p53-p21 axis [61].

**Table 3:** MEK/ERK/SMAD1 signaling pathway in cancer biology.

Aspect	Details
Pathway Components	MEK, ERK, SMAD1, within the transforming growth factor-beta (TGF-β) pathway [62].
Influences on Cancer	Tumorigenesis, tumor growth, invasion, metastasis, and angiogenesis [62].
Cellular Processes Affected	Cell cycle progression, Epithelial–Mesenchymal Transition (EMT), tumor invasion, metastasis, angiogenesis [62,63].
Therapeutic Targeting	Small-molecule inhibitors targeting MEK or ERK show efficacy in preclinical and clinical studies. The TGF-β pathway, including SMAD proteins, is considered a potential target for cancer therapy [63].
Challenges in Targeting	Context-dependent effects, varied response across different cancer types, intricate crosstalk with other signaling pathways.

**Table 4:** MEK/ERK pathway in cell processes.

Cellular Process	Details
Cell Growth and Proliferation	Aberrant activation of the MEK/ERK pathway is implicated in driving uncontrolled cell growth through phosphorylation events [72].
Epithelial–Mesenchymal Transition (EMT)	MEK/ERK pathway induces EMT, enhancing migratory and invasive capabilities of cancer cells. SMAD1 contributes to EMT regulation [67].
Metastasis	Associated with promoting tumor invasion, influencing cell adhesion, extracellular matrix remodeling, and cytoskeletal changes. SMAD1 enhances metastasis by regulating genes related to cell motility [70].
Angiogenesis	Implicated in angiogenesis, with ERK influencing the expression of angiogenic factors. SMAD1 contributes to angiogenesis regulation [71].

**Table 5:** MEK/ERK/SMAD1 interplay in specific cancers.

Cancer Type	Interplay Details
Prostate Cancer	PTEN loss activates the PI3K/AKT/mTOR pathway, leading to the upregulation of GLO1 and GLO2 within the glyoxalase system via the PI3K/AKT/mTOR/p-PKM2(Y105)/ERa axis, and it is involved in prostate cancer progression [82].
Non-Small-Cell Lung Cancer	Glo2 is implicated in the proapoptotic effects of Oleuropein [56].
Breast Cancer	GLO1 silencing results in endogenous dicarbonyl stress, activating the MEK/ERK/SMAD1 pathway. The functional link to a pro-metastatic phenotype is uncovered [85].

**Table 6:** Correlation of Glo1 overexpression with clinical features in breast cancer patients.

Clinical Feature	Correlation with Glo1 Overexpression
Lymph Node Metastasis	Significant positive correlation, indicating a potential role of Glo1 in promoting lymphatic spread in breast cancer [90].
Lymphovascular Invasion	Elevated levels of Glo1 are associated with an increased likelihood of lymphovascular invasion, suggesting its involvement in tumor progression [92].
Tumor Grade	Positive correlation with higher tumor grade, emphasizing Glo1’s association with more aggressive and poorly differentiated tumors [93].
TNM Stage	Advanced TNM stages show a strong association with Glo1 overexpression, suggesting its role as a prognostic indicator for disease progression [93].
Prognostic Implications	Glo1 overexpression was independently linked to shorter overall survival and recurrence-free survival, highlighting its potential as a prognostic marker [94].

**Table 7:** Therapeutic implications and experimental approaches targeting Glo1 in breast cancer.

Therapeutic Aspect	Experimental Approaches and Findings
Chemotherapeutic Resistance	Upregulation of Glo1 linked to resistance, experimental Glo1 inhibitors, like Troglitazone, demonstrated efficacy in restoring sensitivity to doxorubicin [96].
Radiotherapy Response	Glo1 overexpression is associated with poor response; potential targets include Glo1 inhibitors and modulation of MGO levels to enhance radiotherapy outcomes [94].
Distant Metastasis	Cooperative involvement of Glo1 and PKCλ; inhibitors (TLSC702 and aurothiomalate) show promise in suppressing late-stage breast cancer progression [102].
Dualistic Role of Glo1 in Tumor Growth and Metastasis	Silencing Glo1 led to elevated MGO levels, promoting tumor growth and metastasis; this underscores the dual nature of Glo1’s impact on breast cancer progression [104].

**Table 8:** Overview of ovarian cancer and diagnostic challenges.

Aspect	Details
Prevalence and Lethality	Most lethal among female reproductive tract malignancies. In the United States, despite not being as prevalent, it asserts prominence as the most lethal. The five-year survival rate is around 47% [106,109].
Diagnosis and Treatment Approach	Gold standard diagnosis relies on pathological biopsy. Late-stage diagnoses are common due to the absence of effective screening methods. Standard treatment involves cytoreductive surgery and postoperative adjuvant chemotherapy [107,108].
Diagnostic Biomarkers	Existing biomarkers (CA-125, HE4) show promise but have limitations. Urgent need for novel biomarkers for early detection [111].

**Table 9:** Emerging therapeutic targets and biomarkers in ovarian cancer.

Biomarker/Therapeutic Target	Details
Receptor for Advanced Glycation End Products (RAGE)	Upregulated in ovarian cancer tissue compared with normal tissue. Correlation with poor clinicopathological features. Potential as a biomarker with high sensitivity and specificity [114].
Glyoxalase 1 (Glo1)	Elevated expression in FH-OSE cells associated with ovarian cancer occurrence and progression. Overexpression in invasive ovarian cancers. Potential marker for early detection and therapeutic target. Mechanisms and exact roles in ovarian cancer require further research [117].
